# Does Continuous Positive Airway Pressure Therapy in Patients with Obstructive Sleep Apnea Improves Uric Acid? A Meta-Analysis

**DOI:** 10.1155/2019/4584936

**Published:** 2019-09-15

**Authors:** Qingshi Chen, Guofu Lin, Lida Chen, Jiefeng Huang, Yaping Huang, Ping Li, Mengxue Chen, Qichang Lin

**Affiliations:** ^1^Department of Respiratory and Critical Care Medicine, The First Affiliated Hospital of Fujian Medical University, No. 20 Chazhong Road, Taijiang District, Fuzhou 350005, China; ^2^The Second Affiliated Hospital of Fujian Medical University, No. 34 Zhongshan North Road, Licheng District, Quanzhou 362000, China; ^3^Department of Respiratory and Critical Care Medicine, Zhangzhou Affiliated Hospital of Fujian Medical University, No. 59, Shenglixi Road, Xiangcheng District, Zhangzhou 363000, China

## Abstract

**Purpose:**

The efficacy of obstructive sleep apnea (OSA) therapy with continuous positive airway pressure (CPAP) on uric acid (UA) yielded conflicting results. This meta-analysis was performed to assess whether OSA treatment with CPAP could reduce UA levels.

**Methods:**

The Web of Science, Cochrane Library, Embase, and PubMed were searched before March 2019. Information of patients, study design, and pre- and post-CPAP treatment of UA was utilized for analysis. The overall effects were analyzed via the standardized mean difference (SMD) with a 95% confidence interval (CI). Five studies were obtained and the meta-analysis was performed using Stata 12.0 and Review Manager 5.2.

**Results:**

A total of 5 studies with 6 cohorts (2 RCT and 3 observational studies) involving 270 patients were pooled into meta-analysis. There was no change of UA levels before and after CPAP treatment in OSA patients (SMD = ‐0.20, 95% CI: -0.78 to 0.37, *Z* = 0.69, *p* = 0.49). Subgroup analysis showed that the outcomes were not affected by age of patients, gender distribution, baseline body mass index, daily duration, duration of CPAP treatment, sample size, and study design.

**Conclusions:**

This meta-analysis revealed that CPAP treatment has no effect on UA in OSA patients. Further well-designed, large-scale randomized controlled trials are required to address this issue.

## 1. Introduction

Obstructive sleep apnea (OSA) is characterized by recurrent hypopnea or apnea during sleep, which leads to sleep fragment or intermittent hypoxia (IH). OSA has been estimated to occur in 17% of women and 34% of men [1], affecting approximately 250 to 500 million people worldwide [2]. OSA patients are at an increased risk for cardiovascular disease, diabetes mellitus, obesity, metabolic dysregulation, accidents, and all-cause mortality [3–6] and are a huge public health burden [7].

The IH observed in OSA patients may have an impact on the purine metabolic approach, increasing the formation of adenosine triphosphate (ATP) degradation and the excretion of intermediates of the purines, which ultimately results in an overproduction of uric acid (UA). As a matter of fact, hyperuricemia and increased nocturnal urinary UA excretion are universal features in OSA [8]. An independent association between UA levels and OSA has also been found in clinical trials [9]. Meanwhile, Chou et al. [10] reported that hyperuricemia was found in about 56% of OSA patients. Other studies also indicated that there was an association between increased uric acid levels and the presence of OSA [11, 12]. Moreover, UA also had been recently found as an independent risk factor for cardiovascular disease in OSA patients [12, 13].

CPAP is currently recognized as a main accepted treatment for OSA. Previous studies indicated that treatment with CPAP treatment can reduce the risk of cardiovascular events associated with OSA [14]. However, whether the levels of UA can be ameliorated or not by CPAP remains inconclusive. The main goal of the current meta-analysis was to quantitatively assess the efficacy of CPAP therapy on UA levels among patients with OSA.

## 2. Methods

### 2.1. PRISMA Statement

This meta-analysis was conducted according to the recommendations of the PRISMA (Preferred Reporting Items for Systematic Reviews and Meta-Analyses statement) [15].

### 2.2. Literature Search

The PubMed, Embase, Cochrane Library, and Web of Science were searched before March 3, 2019, for related literature. All searches were conducted by combining free-text and MeSH terms, and the following terms were performed: “sleep apnea sleep apnea OR sleep apnea OR apnea OR sleep apnoea” and “uric acid OR urate” and “continuous positive airway pressure or CPAP”. No language restrictions were imposed. In addition, the reference lists of the included articles were manually screened for additional relevant studies. Two independent assessors identified the eligible studies.

### 2.3. Inclusion and Exclusion Criteria of the Literature

Studies that fulfilled the following criteria were considered eligible: (1) Patients in all studies were diagnosed with OSA. (2) The intervention was an application of CPAP. (3) The article must report the value of UA both before and after CPAP. (4) The studies had sufficient data to perform a meta-analysis. (5) Abstracts, reviews, case reports, letters to the editor, conference articles, animal studies, and non-English studies were excluded.

### 2.4. Data Extraction

Two investigators assessed the eligible studies independently. Inconsistent decisions were resolved through a consensus with a third researcher. The variables extracted from each article included the following: first author, publication date, nation of article, the number of patients, gender distribution, mean age of patients, CPAP duration, mean daily duration, AHI, body mass index (BMI), study design, and UA values before and after CPAP therapy.

### 2.5. Statistical Analysis

Statistical analyses were performed using the Cochrane Review Manager software (version 5.2) and STATA statistical software version 12.0. The pooled estimate of the SMD and 95% CI was calculated. Statistical heterogeneity was assessed based on the Chi^2^ and the *I*^2^ statistics, with *I*^2^ > 50% indicating significant heterogeneity. A random effects model was applied to combine an effect size if *I*^2^ > 50%; otherwise, a fixed effects model was used. Furthermore, subgroup analysis of CPAP treatment time (≤3 months and >3 months), age of patients (≤50 year and >50 year), mean daily duration (<4 h and ≥4 h), and sample size (≤30 and >30) was performed to identify the possible sources of heterogeneity. Sensitivity analysis was performed by omitting one study in each turn, and publication bias was assessed using both the “Begg test” and the “Egger test.” Statistical significance was reached if a *p* < 0.05 was obtained.

## 3. Results

### 3.1. Searching Results

**Figure 1:**
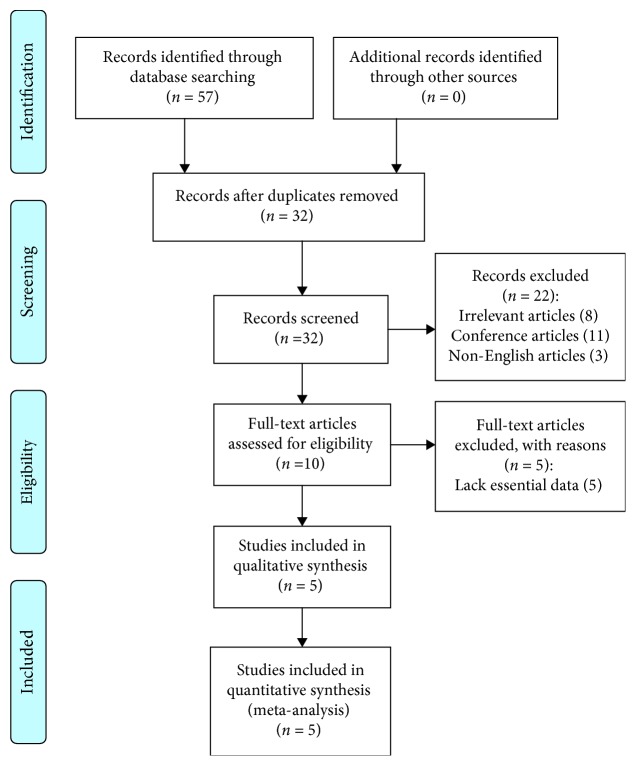
Flow diagram of the literature search.

Our search identified 57 references after excluding duplicates. After a further review of the titles and abstracts, 24 studies were considered to be potentially relevant. Among the 24 studies, 19 studies were subsequently excluded (see detail in Figure 1). Finally, 5 studies [8, 16–18] were included in the meta-analysis. Two of them were RCT [16, 18]; three were observational study. The detailed steps of the literature search were shown in Figure 1.

### 3.2. Characteristics of Included Studies

A total of five articles including six cohorts with 270 patients met the inclusion criteria and were included in this meta-analysis. Two of them were randomized clinical trials (RCTs) [16, 18]; the other studies were observational [8, 17, 19]. One study [19] reported results separately for the good compliance group (mean CPAP use ≥4 h/night) and the poor compliance group (mean CPAP use <4 h/night). Four studies [8, 17–19] defined OSA based on the apnea-hypopnea index (AHI), while Prudon et al.'s study [16] defined OSA based on the oxygen desaturation index (ODI). The information of author, publication date, nation, sample size, gender distribution, inclusion criteria, daily duration, CPAP duration, and study design of each study is summarized in Table 1. The information of mean age, AHI, BMI, and UA of each study is presented in Table 2. Two RCT studies are summarized separately in Table 3.

### 3.3. Pooled Analysis

**Figure 2:**
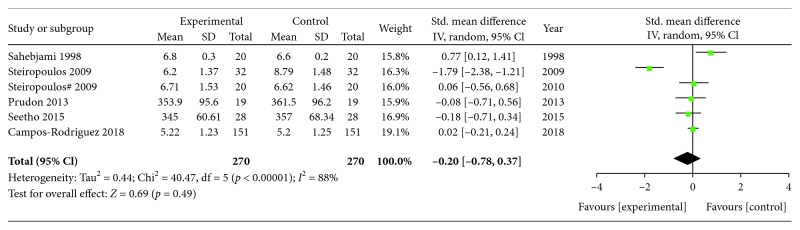
Forest plot for the change in uric acid before and after CPAP treatment.

**Figure 3:**
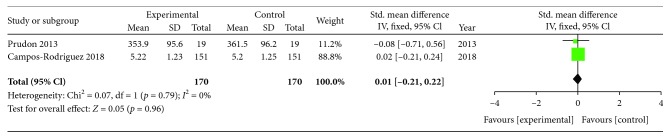
Forest plot for the change in uric acid between the CPAP treatment group and the control group in two RCTs.

The heterogeneity test showed that there were significant differences among the included studies (Chi^2^ = 40.47, *p* = 0.00001; *I*^2^ = 88%). Therefore, an effect size was combined using a random effects model. After pooling the data with meta-analysis, no significant difference in UA in OSA patients was observed before and after CPAP treatment (SMD = ‐0.20, 95%CI = ‐0.78 to 0.37, *Z* = 0.69, *p* = 0.49) (Figure 2). A similar result (SMD = ‐0.11, 95%CI = ‐0.28 to 0.06, *Z* = 1.27, *p* = 0.20) was also obtained based on a fixed effects model. Meta-analysis of the two RCTs showed that UA has no change in the CPAP group when compared with the control group (SMD = 0.01, 95%CI = ‐0.21 to 0.22, *Z* = 0.05, *p* = 0.96, random effects model) (Figure 3).

### 3.4. Subgroup Analysis and Sensitive Analysis

**Figure 4:**
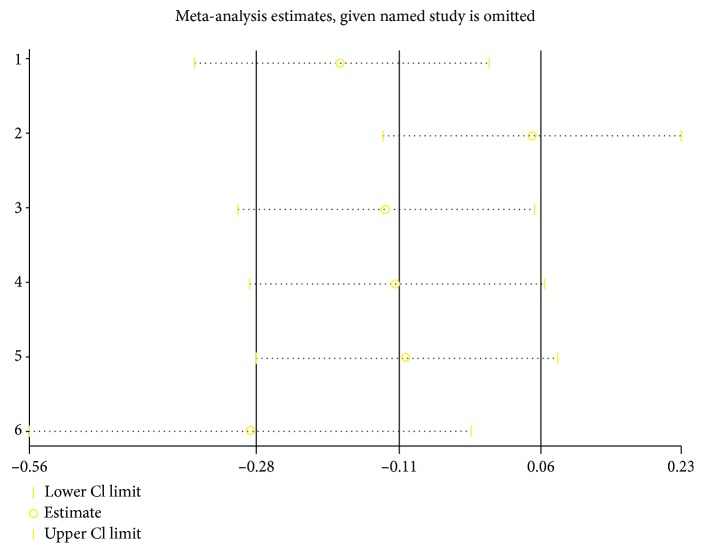
Sensitivity analysis of the included studies. CI: confidence interval.

Sensitivity analysis showed that deleting any one of the studies at a time did not influence the overall result of the pooled analysis (Figure 4). Considering the effectiveness of CPAP could be affected by many factors, subgroup analyses were performed based on factors such as age (≤50 and >50), baseline BMI (<35 and ≥35), daily duration (<4 h and ≥4 h), CPAP therapy duration (≤3 month and >3 month), gender distribution (women and male), sample size (≤30 and >30), and study design. The results demonstrated that differences in age, BMI, daily duration, CPAP duration, sample size, and study design did not influence CPAP efficacy (Table 4).

### 3.5. Publication Bias

**Figure 5:**
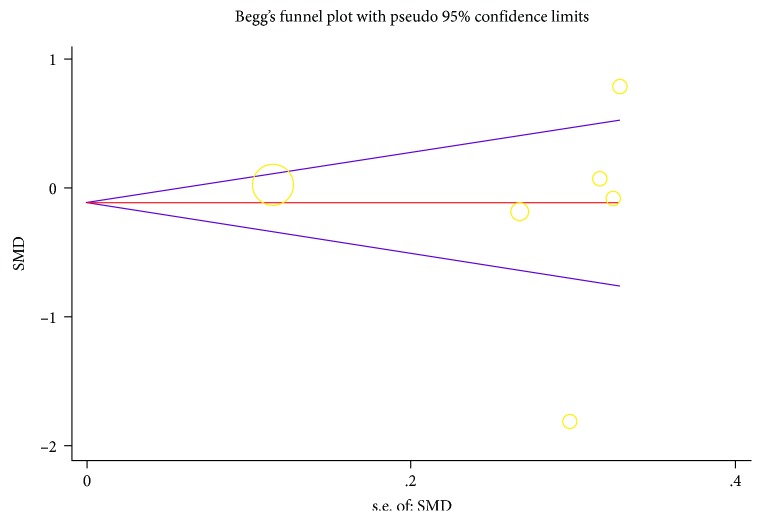
Funnel plots for assessing publication bias of studies included. SMD: standardized mean difference, SE: standard error.

Figure 5 shows that publication bias seems to exist, however, both the Begg and Egger tests proved that no publication bias existed in the present study (*p* = 0.707 and 0.846, respectively).

## 4. Discussion

To our best knowledge, this was the first meta-analysis evaluating the effect of CPAP on UA in OSA patients. The results of the present meta-analysis of 5 studies and 6 cohorts indicated that CPAP therapy exerted no impact on UA in patients with OSA. Subgroup analysis also did not find a discrepant effect of CPAP therapy on UA inpatients with OSA.

Increasing evidence supported that the serum UA level is high in OSA patients [11]. Similarly, all patients with OSA, included in the present meta-analysis, showed a high baseline uric acid level. A growing body of favorable evidence has identified the association between a high uric acid level and OSA with multiple associated mechanisms. First, repetitive intermittent hypoxia, a main character of OSA, is associated with the increased production of reactive oxygen species (ROS) and can change the integrity of cellular metabolic processes during episodes of OSA [8]. Inadequate oxygen supplementation can impair the formation of ATP. Finally, it will lead to an overproduction of uric acid. Furthermore, lactic acid generated during hypoxia can bring about the urinary excretion of lactate, which is in favor of the absorption of urate in the proximal tubule [20]. Relief of hypoxic episodes, either with CPAP treatment or with weight reduction, has been demonstrated to reduce serum uric acid levels in OSA patients [19]. Finally, owe to long-term sleep disturbance, hypoxia causes a decrease of ATP production in tissue cells, and the inhibition of phosphoribosyl amide transferase is weakened, and an increase in purine production results in a high blood UA level.

Oxidative stress, one of the results of intermittent hypoxia during OSA, is also a common pathway to the production of UA, and it thus can be reflected in UA levels [21]. Meanwhile, oxidative stress is related to cardiac and vascular defects resulting in hypertension and atherosclerosis [22, 23]. In this sense, hyperuricemia, an independent predictor of death in patients with cardiovascular disease [24], is usually found in OSA patients. As early as in 1998, Sahebjami [8] had shown that uric acid excretion was increased in OSA patients and normalized after CPAP treatment, which most likely reflected an association between hyperuricemia and OSA. High serum UA levels are also independently associated with an increased risk of cardiovascular morbidity and mortality. Some evidence suggests that hyperuricemia is associated with cardiovascular disease in patients with OSA [12].

Hyperuricemia was deemed as an independent predictor of death in patients with a high risk of cardiovascular disease [24]. Sahebjami [8] had shown that UA excretion was increased in OSA patients and normalized after CPAP, most likely demonstrating an association between OSA and hyperuricemia. In 2019, Shi's team [25] conducted a meta-analysis of the association between UA and OSA and also reported that OSA may be a potential risk for hyperuricemia. The possible mechanisms behind the association between OSA and hyperuricemia were mainly related to intermittent hypoxia during OSA. As discussed above, the mechanisms included the process of ROS, ATP, oxidative stress, and so on.

CPAP is known as the first choice for OSA. Previous reports have confirmed the efficacy of CPAP therapy on the risk of cardiovascular events related to OSA; however, whether serum UA can be changed by CPAP therapy in OSA patients remains controversial. In the present meta-analysis, all the 3 self-control trials with 4 cohorts suggested that CPAP can reduce UA in the OSA patients, while the remaining 2 RCTs showed that there was no influence of CPAP on serum UA levels. Furthermore, further subgroup analysis indicated that the results did not show change when subdivided by age, BMI, daily duration, CPAP duration, sample size, and study design. Meanwhile, we performed sensitivity analysis to omit each including study; the pooled SMD remained unchanged in the meta-influence analysis.

We acknowledge that there are various factors possibly causing hyperuricemia in OSA patients. UA is associated with the magnitude of AHI, SaO_2_ dips, the number of arousals, blood pressure, BMI, age, cholesterol, and triglycerides in patients with OSA [9]. However, the results of the meta-analysis exclude to date that CPAP decreases UA levels. Given the relationship between UA and cardiovascular risk, clinicians should take into account other therapeutic measures, e.g., weight loss, drugs, and exercise in the management of UA in OSA patients.

Our meta-analysis has its own advantage. Firstly, pooling of the data from all eligible clinical studies yielded more precise and reliable outcomes than that from an individual study. Secondly, two RCTs with a relatively high Jadad score were included in our meta-analysis; the consistency of negative results strengthened our conclusion. Thirdly, there was no significant evidence of publication bias in our meta-analysis. Fourthly, sensitivity analysis and subgroup analysis were performed in our study; both the results did not reach a statistical significance.

However, there are several intrinsic limitations of this study. Firstly, most of the included studies were an observational study; only two RCTs with pre- and posttreatment data rather than treatment and control groups data were drawn. The reliability of our conclusions may be weakened due to the property of the design. Secondly, the relatively small number of studies restricts the extrapolation of our conclusion; additional large-scale, well-designed, and long-term RCTs are required. Thirdly, some potential confounders including BMI, diets, and urate-lowering therapy during follow-up were difficult to fully adjust. The results of the present study should not lead to disregarding the well-recognized role of diabetes, hypertension, and renal function. Fourth, treatment periods of these trials were various, which may contribute to the heterogeneity of this meta-analysis. Fifth, only papers published in English were included; this may cause potential publication bias.

## 5. Conclusions

In summary, our meta-analysis demonstrated that CPAP treatment does not improve the UA levels in OSA patients. Further large-scale well-designed RCTs with long-term follow-up are needed to address this issue.

## Figures and Tables

**Table 1:** Characteristics of included studies (5 studies, 6 cohorts).

First author	Year	Nation	Sample size/male	Inclusion criteria	Daily duration (h/night)	CPAP duration	Study design
Sahebjami	1998	USA	20/20	OSAHS (NR)	NR	1 D	SCT
Steiropoulos (≥4 h)	2009	Greece	32/32	AHI ≥ 5	≥4 h	6 M	SCT
Steiropoulos (<4 h)	2009	Greece	20/20	AHI ≥ 5	<4 h	6 M	SCT
Prudon	2013	UK	19/19	ODI > 10, with ESS ≥ 9	<4 h	3 M	RCT
Seetho	2015	UK	28/16	AHI ≥ 5	≥4 h	14 M	SCT
Campos-Rodriguez	2018	Spanish	151/0	AHI ≥ 15	≥4 h	3 M	RCT

Abbreviation: CPAP: continuous positive airway pressure, AHI: apnea-hypopnea index, ODI: oxygen desaturation index, ESS: epworth sleepiness scale, M: month, D: day, h: hour, NR: not reported, SCT: self-control trials, RCT: randomized controlled trials.

**Table 2:** Details of included studies.

First author	Age (years)	AHI (events/h)	ODI (events/h)	ESS score	BMI (kg/m^2^)	Pre-UA	Post-UA
Sahebjami	55.1 ± 2.8	53 ± 6	NR	NR	36.4 ± 5.0	6.6 ± 0.2 (mg/dl)	6.8 ± 0.3 (mg/dl)
Steiropoulos (≥4 h)	45.63 ± 10.73	61.12 ± 28.29	63.53 ± 28.47	11.91 ± 5.59	≥35	8.79 ± 1.48 (mg/dl)	6.2 ± 1.37 (mg/dl)
Steiropoulos (<4 h)	46.6 ± 9.91	50.71 ± 26.23	49.38 ± 24.44	9.55 ± 5.62	33.3 ± 6.5	6.62 ± 1.46 (mg/dl)	6.71 ± 1.53 (mg/dl)
Prudon	58.1 ± 10.6	NR	33.8 ± 21.9	14.5 ± 3.5	39.6 ± 1.9	361.5 ± 96.2 (*μ*mol/l)	353.9 ± 95.6 (*μ*mol/l)
Seetho	50 ± 7	≥5	NR	5.42 ± 6.25	34.58 ± 8.92	357 ± 68.34 (*μ*mol/l)	345 ± 60.61 (*μ*mol/l)
Campos-Rodriguez	58.8 ± 9.6	36.54 ± 19.23	35.91 ± 19.76	10.2 ± 4.2	33.55 ± 4.51	5.20 ± 1.25 (mg/dl)	5.22 ± 1.23 (mg/dl)

Values are mean ± SD. Abbreviation: AHI: apnea-hypopnea index, ODI: oxygen desaturation index, ESS: epworth sleepiness scale, NR: not reported, BMI: body mass index, UA: uric acid.

**Table 3:** Characteristics of two randomized controlled trials.

First author	Year	Study design	Treatment group				Control group				Jadad score
			Samples	Pre-UA	Post-UA	*p* value	Samples	Pre-UA	Post-UA	*p* value	
Prudon	2013	Parallel	19	361.5 ± 96.2 (*μ*mol/l)	353.9 ± 95.6 (*μ*mol/l)	0.9	19	412.6 ± 91.2 (*μ*mol/l)	406.4 ± 90.8 (*μ*mol/l)	0.9	3
Campos-Rodriguez	2018	Parallel	151	5.20 ± 1.25 (mg/dl)	5.22 ± 1.23 (mg/dl)	0.702	156	5.02 ± 1.26 (mg/dl)	5.07 ± 1.31 (mg/dl)	0.208	5

**Table 4:** The results of subgroup analyses.

Subgroup	No. of study and patients	Heterogeneity			SMD			
		Chi^2^	*p*	*I* ^2^ (%)	SMD	95% CI	*Z*	*p*
Age								
≤50	3/80	22.70	≤0.001	91	-0.64	-1.76 to 0.48	1.12	0.26
>50	3/190	4.93	0.09	59	0.18	-0.26 to 0.63	0.81	0.42
BMI								
<35	3/203	32.64	≤0.001	94	-0.56	-1.65 to 0.53	1.00	0.32
≥35	3/67	5.54	0.06	64	0.15	-0.43 to 0.73	0.51	0.61
Daily duration								
<4 h	2/39	0.09	0.76	0	-0.01	-0.45 to 0.44	0.03	0.97
≥4 h	3/211	31.97	≤0.001	94	-0.63	-1.64 to 0.38	1.22	0.22
CPAP duration								
≤3 month	3/190	4.93	0.09	59	0.18	-0.26 to 0.63	0.81	0.42
>3 month	3/80	22.70	≤0.001	91	-0.64	-1.76 to 0.48	1.12	0.26
Gender								
Women	2/163	0.21	0.65	0	0.00	-0.22 to 0.22	0.02	0.99
Male	5/107	37.43	≤0.001	89	-0.25	-1.12 to 0.62	0.57	0.57
Sample size								
≤30	4/87	5.57	0.13	46	0.12	-0.29 to 0.53	0.58	0.56
>30	2/183	31.96	≤0.001	97	-0.87	-2.64 to 0.91	0.96	0.34
Study design								
SCT	4/100	36.98	≤0.001	92	-0.29	-1.33 to 0.75	0.55	0.58
RCT	2/170	0.07	0.79	0	0.01	-0.21 to 0.22	0.05	0.96

AHI: apnea-hypopnea index, BMI: body mass index, SMD: standardized mean difference, CI: confidence interval, SCT: self-control trials, RCT: randomized controlled trials.
